# Intra- and Inter-host Transmission Dynamics of SARS-CoV-2 Through Viral Load Data Analysis

**DOI:** 10.7759/cureus.82955

**Published:** 2025-04-24

**Authors:** Sofia Liossi, Evangelos Tsiambas, Sotirios Maipas, Marilena Argyropoulou, Effie G Papageorgiou, Andreas C Lazaris, Nikolaos Kavantzas

**Affiliations:** 1 First Department of Pathology, School of Medicine, National and Kapodistrian University of Athens, Athens, GRC; 2 Department of Cytopathology, 417 Army Equity Fund Hospital (NIMTS), Athens, GRC; 3 Department of Pathology, 417 Army Equity Fund Hospital (NIMTS), Athens, GRC; 4 Department of Biomedical Sciences, University of West Attica (UniWA), Athens, GRC; 5 First Department of Pathology, School of Medicine, National and Kapodistrian University of Athens, Athens General Hospital "Laikon", Athens, GRC

**Keywords:** cycle threshold, disease severity, sars-cov-2, transmission dynamics, viral load

## Abstract

Polymerase chain reaction (PCR) tests are the gold standard for confirming COVID-19. Test results provide the cycle threshold (Ct) value, which is correlated to the patient's viral load as well as hematological and biochemical parameters. The purpose of this study is to analyze the transmission dynamics of selected SARS-CoV-2 variants, both within hosts and between hosts, through statistics and data analysis of the Ct values and other metrics. Demographics data and Ct values from 1,041 patients with COVID-19 were collected and correlated with epidemiological indices, such as the positivity rate, hospitalizations, and deaths for each major wave of the pandemic, in Greece. The analysis showed that higher viral loads coincide with rising pandemic waves, while lower loads are observed during periods of decline. Notably, among all variants analyzed, the Delta variant, observed in mid-2021, exhibited the highest viral load values, which were associated with increased hospitalizations and mortality, despite a relatively low positivity rate. Consequently, variables associated with inter-host transmission dynamics show a significant correlation with those pertaining to intra-host dynamics. This correlation opens up the potential for predicting disease severity and forecasting the trajectory of the pandemic based on patient-related and other variables through data analysis. The analysis revealed that variations in Ct value yield valuable insights into the evolution of the pandemic and the risk stratification of patients. The study highlights that statistical measures derived from Ct values can provide insights into both intra-host and inter-host transmission dynamics, potentially supporting risk assessment and public health responses.

## Introduction

During the COVID-19 pandemic, many mathematical, statistical, and machine learning models were developed to simulate the dynamics of viral transmission or to predict the evolution of the pandemic. The development of models that describe the viral or transmission dynamics uses or relies on data such as the number of new cases, deaths, positivity rates, hospitalized patients, etc. The measurements of these metrics often contain errors, which, when combined with errors inherent in the model, lead to uncertain prediction results [1]. For example, susceptible-exposed-infected-recovered (SEIR)-type models require many parameters that are often estimated or approximated. Errors in these parameters may be significant, and since SEIR-type models rely on numerous parameters, this fact increases the uncertainty of the predictions [2,3]. If we could better understand the evolution of a pandemic wave using more reliable indicators or simple approaches, we may achieve more accurate predictions.

Moreover, most models depend on initial conditions or past data to generate predictions. However, these data, such as the number of cases or deaths, are dynamically evolving. Such changes are often not incorporated into models, resulting in a lack of real-time adjustment. While mathematical models can highlight trends in certain variables, they often fail to precisely determine the absolute values of these parameters [4]. In addition, their predictions may be complex, based on assumptions [4], and not always accurate [5]. Many models were developed during the pandemic to predict its evolution and patient outcomes; however, their effectiveness was often limited due to reliance on uncertain assumptions, delayed data availability, or the inability to incorporate real-time changes [4-6]. Simpler approaches, on the other hand, like indicator analysis, tend to be more user-friendly and may offer comparable reliability [7]. A significant number of COVID-19 models produce forecasts that are similar to those generated by simple linear trends or static baseline models [6]. This highlights the value of exploring reliable and easily interpretable indicators. One of the aims of the present study is to examine the cycle threshold (Ct) value as a potential indicator for understanding the evolution of the pandemic.

Viral load is a key variable used in fitting models of viral dynamics within the hosts [8]. Predicting changes in viral load during illness, combined with other variables, simulates disease progression and could help determine the therapeutic options for the patient [9]. Polymerase chain reaction (PCR) tests are the gold standard for detecting COVID-19. The Ct value, identified from these tests, is associated with viral load as well as other hematological and biochemical indicators. In many clinical trials, the Ct value has been correlated with disease severity, progression, recovery, and infectivity [10,11]. The severity of disease associated with high viral load may be explained by its positive correlation with inflammatory markers or immune response cytokines [12].

Apart from virus characteristics, models also aim to closely capture real-world conditions, such as public health measures, social behaviors, and the overall progression of the pandemic. To achieve this, they often develop specific terms or indicators. The application of non-pharmacological interventions (NPIs), such as restrictive measures, has significant implications for pandemic control. During the pandemic, indicators and metrics were developed to describe these interventions and their impacts, such as the stringency index. The Oxford Coronavirus Government Response Tracker (OxCGRT) developed the stringency index, an indicator that describes and quantifies the strictness of restrictive measures. The stringency index is an average score of nine response measures, including school or workplace closures, restrictions on public gatherings, etc. The index ranges from 0 to 100, with the highest values corresponding to the strictest responses [13,14]. This index was incorporated in this study to capture population behavior during the pandemic.

In this study, we aim to investigate the relationships between basic variables related to viral loads, new cases or deaths, hospitalizations, intensive care unit (ICU) hospitalizations, positivity rate, reproduction rate, and others commonly used in mathematical modeling. The study aims to retrospectively produce simple insights into the pandemic evolution in Greece and identify key variables or correlations to predict the evolution of the next possible pandemic. It specifically explores whether viral load data, reflected through Ct values, can be linked to transmission dynamics, both intra-host and inter-host, and across different waves of the pandemic. In the future, the choice of reliable indicators for monitoring a pandemic, as well as assessing disease severity and risk stratification, must be clarified to facilitate the operation of health systems.

## Materials and methods

In this study, we investigate whether there is a correlation between the Ct values of patients with COVID-19 and the evolution of the pandemic by applying basic statistical methods of data analysis. The advantage of this approach is that the Ct value is a relatively valid and easily accessible indicator. Therefore, we aim to link data relating to the dynamics of the SARS-CoV-2 virus within the host to its dynamics between hosts. For this purpose, we statistically analyzed data for COVID-19 patients, including demographic characteristics, such as gender and age, Ct value, and whether the patient was hospitalized in the ICU or not, during the periods when the Alpha, Delta, and Omicron variants predominated in the country, but also an initial period corresponding to the first variants that appeared. Additionally, we examined whether the Ct values are related to disease severity and, as a result, can be used as a biomarker for the progression of the disease in the patient. Therefore, by focusing on a single variable, we attempted to draw conclusions about dynamics both within and between hosts. Approval for the study was acquired from the Bioethics and Deontology Committee of the School of Medicine at the National and Kapodistrian University of Athens, Greece (approval number: 564; date: 11/19/2021). The dataset consisted of fully anonymized, retrospective patient data collected as part of routine diagnostic procedures. All confidentiality and data protection measures were strictly observed. The data were analyzed using Python, executed on a MacBook Air (Apple M1 system, 16 GB RAM), using the libraries Pandas, NumPy, SciPy, and Seaborn for data processing and visualization.

**Figure 1:**
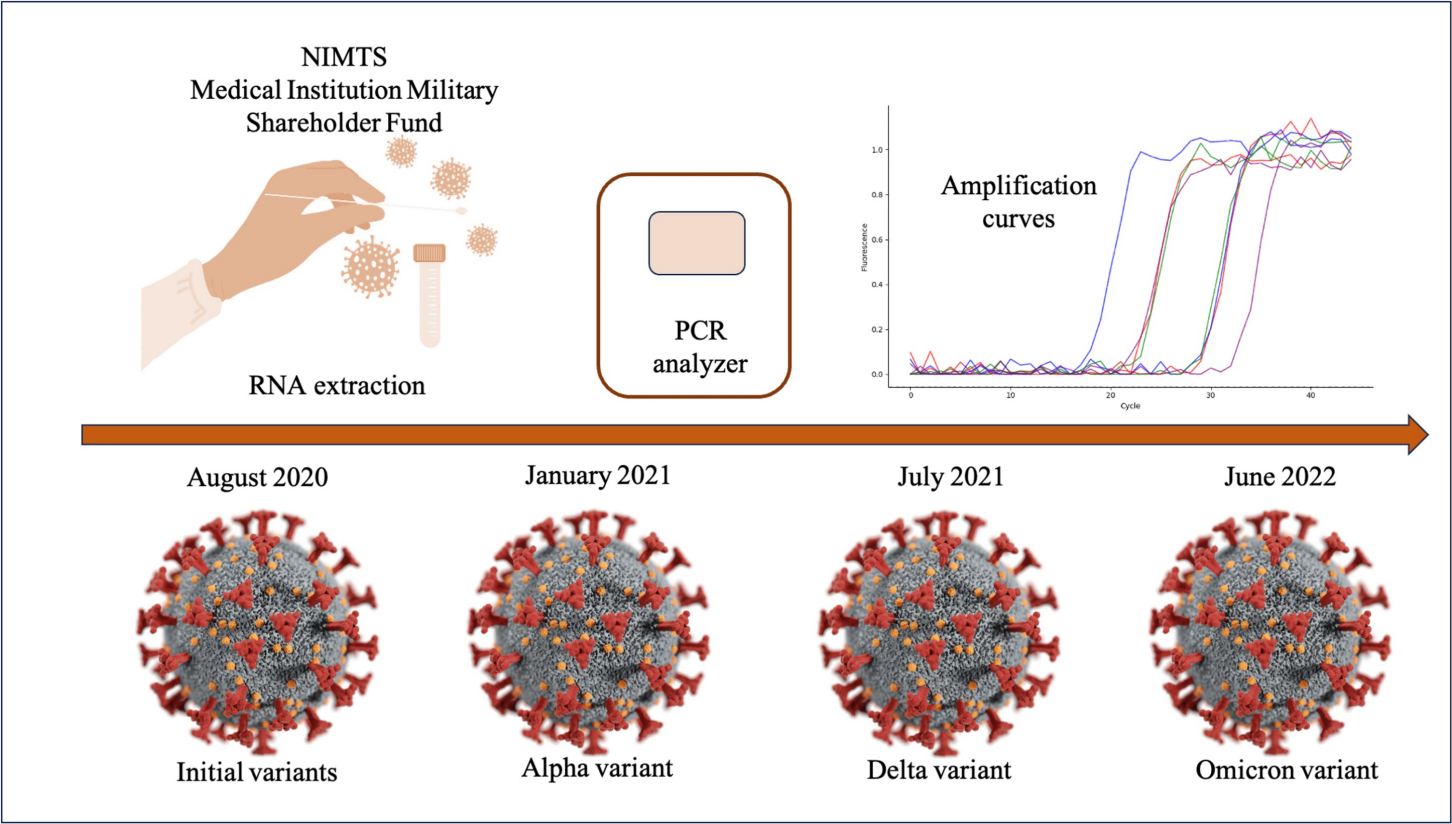
Graphical presentation of the period of the study and the variants of SARS-CoV-2 NIMTS: 417 Army Equity Fund Hospital; PCR: polymerase chain reaction Source: Authors' own work (graph) using Pixabay

From January 2021 to July 2021, the Alpha variant emerged in Greece, followed by the Delta variant, which remained dominant until the end of 2021, followed by the Omicron variant and its subvariants in the next period. From August 2020 to June 2023, 20,435 PCR tests were carried out at the 417 Army Equity Fund Hospital (NIMTS) Medical Institution Military Shareholder Fund, with 1,765 positive results (8.6%). The samples used in the study were obtained from nasopharyngeal swabs, which were collected as part of routine diagnostic PCR testing at the NIMTS. Samples obtained via bronchoalveolar lavage (BAL) were excluded. For each positive case, age, gender, and information on possible hospitalization in an ICU were recorded. A sample was considered positive when the Ct value was below 40. Repeated tests from the same patient were excluded from the study. The data derive from a retrospective hospital file (NIMTS) and concern exclusively anonymized cases. The data were collected and grouped by variant (Alpha, Delta, or Omicron) from August 2020 to June 2022. The virus variant was not genetically identified for each patient; instead, classification was based on the dominant variant in Greece during the corresponding period, according to official data from the National Public Health Organization, as described below. The sample size in this study consists of 1,041 patients and a pool of variables (Figure 1). In the following analysis, when missing data were present in some variables, a subset of the original sample was selected to ensure the completeness of the data for accurate statistical evaluation. Furthermore, supplementary data were obtained from the [15] database, which provides information on new cases, deaths, positivity rate, reproduction rate, hospitalizations, ICU admissions, and the stringency index.

During the first two years of the COVID-19 pandemic, several initial variants appeared, followed by the Alpha, Delta, and Omicron variants, which prevailed in the country during different periods. The periods used in this study were determined based on data from [16], which indicated the dominant variant during each wave in the country, as published in [17]. In the following sections, viral load data, demographic characteristics of the patients, and epidemiological measures or indicators were used to correlate the dynamics within hosts and between hosts. 

## Results

Ct cycle and demographic characteristics

The statistical analysis examined Ct values as the primary outcome variable in relation to demographic parameters (age, gender), disease severity (ICU admissions and hospitalizations), and the period of variant prevalence (initial, Alpha, Delta, Omicron), as well as commonly used epidemiological indicators such as new cases, deaths, positivity rate, and reproduction rate. Group comparisons were conducted using non-parametric methods (Kruskal-Wallis with post-hoc Dunn's test), and correlation analyses were performed using Spearman's coefficient. Confidence intervals were estimated using the bootstrapping method.

**Figure 2:**
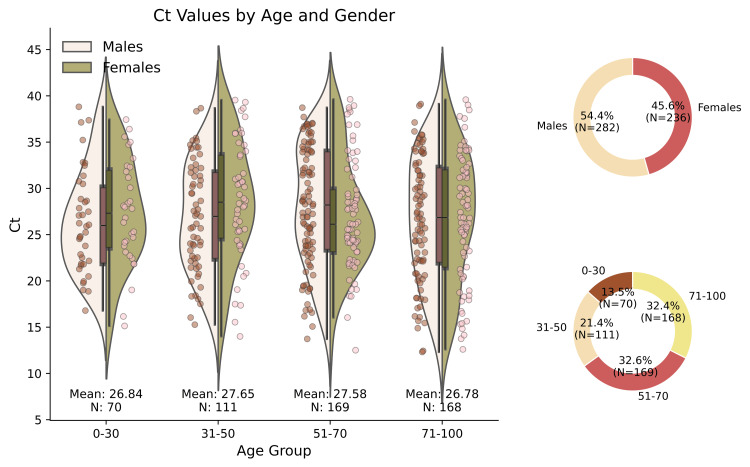
Distribution of Ct values across age groups and genders The violin plots illustrate the distribution of Ct values across different age groups and genders. The horizontal lines show the median Ct value in each boxplot for each gender. The donut charts show the percentage distribution of men and women in the sample, as well as the proportion of participants in each age group (with each segment representing the percentage of individuals in a specific age range). Ct: cycle threshold

The Ct values used to analyze demographic characteristics involve 518 individuals with COVID-19, of whom 236 were women and 282 were men. Of these patients, 13.5% were 30 years old or younger, 21.4% were aged between 30 and 50 years, 32.6% were aged between 50 and 70 years, and 32.4% were 70 years or older. Figure 2 presents the basic statistical information on the Ct value distribution across the 518 patients (all variants), categorized by gender and age groups. Violin plots illustrate the distribution of Ct values across four distinct age groups, categorized by gender. The sample size per group is noted above the graph.

The mean Ct value for the entire sample was 27.24, with the age group means ranging from 26.78 (in those over 70) to 27.65 (in the 30-50 age group). Due to the data distribution, non-parametric tests were used to compare groups. Kruskal-Wallis analysis showed no significant differences in Ct values between genders (p>0.05) or age groups. The same analysis was performed separately for each variant (Alpha, Delta, and Omicron), and the results were consistent, showing no significant differences between genders or age groups. Therefore, the data do not suggest any correlation between viral load and age or sex.

Ct cycle and variant characteristics

**Figure 3:**
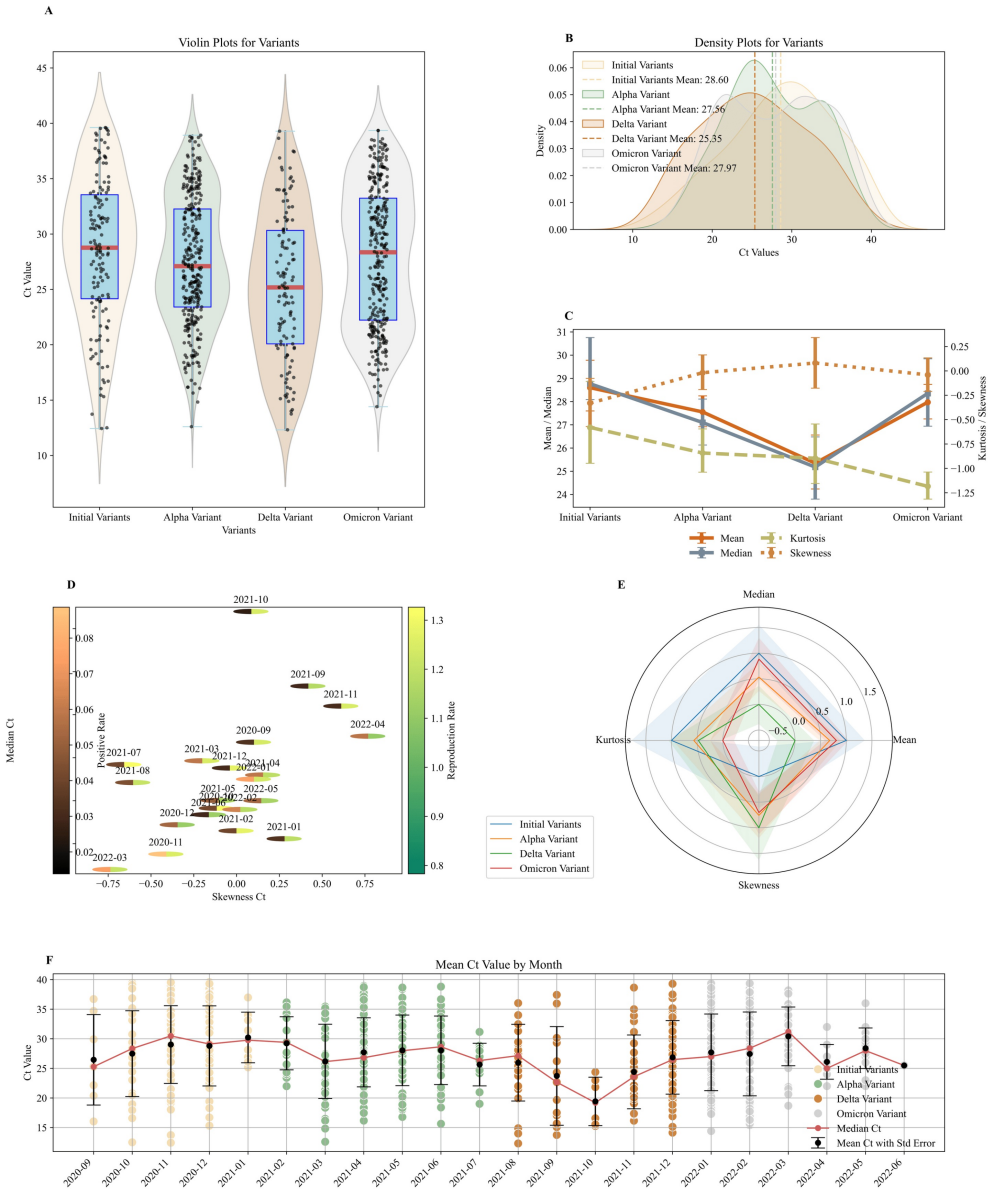
Statistical analysis of Ct values across COVID-19 variants and time periods (A) The violin plots depict the skewness and kurtosis of Ct values for each variant. (B) The density plot illustrates the Ct values across the initial, Alpha, Delta, and Omicron variants. The vertical dashed line indicates the mean value. (C) The chart displays the mean, median, kurtosis, and skewness statistics for each variant. The lines connect the mean values, helping to visualize trends over time, while the error bars represent 95% CIs. (D) The graph illustrates the relationship between skewness and the median Ct value for each month during the study period. The split circles represent the reproduction rate on the right and the positivity rate on the left, with color coding indicating their respective values. (E) The radar chart depicts the normalized values of mean, median, skewness, and kurtosis of each pandemic wave/variant, along with their corresponding CI. (F) The scatter plots display the Ct values by month, from August 2020 to June 2022. The black dots represent the mean value, and the red line connects the median values for each month, with standard errors indicated. Ct: cycle threshold

For each variant, a representative statistical sample was selected from the overall patient record, and the mean Ct values, median, kurtosis, and skewness were calculated. The distribution of the data over the study period was examined to compute statistics and use appropriate metrics in the analysis. The initial variants, Alpha and Delta, approximated a normal distribution, whereas the Omicron variant exhibited a stronger skew. The Kolmogorov-Smirnov test indicated non-normality (p=0.0229) in the Ct values of the Omicron variant (Figure 3B). The mean Ct values were calculated monthly and for each variant (Figure 3F, 3A), along with the other statistical metrics. During the initial variants' period, the highest mean Ct value was observed (μ_initial_=28.60; CI: (27.41, 29.74)), followed by the Omicron variant (μ_omicron_=27.97; CI: (27.21, 28.66)), the Alpha variant (μ_alpha_=27.56; CI: (26.86, 28.17)), and the Delta variant (μ_delta_=25.35; CI: (24.14, 26.44)). The median value follows the same trend, with the lowest value also occurring during the Delta variant period (Q_2,delta_=25.18; CI: (23.8, 26.48)). Confidence intervals were calculated using the bootstrapping method and are presented in Table 1. 

**Table 1:** Statistical measures for the Ct values of the initial, Alpha, Delta, and Omicron variants Ct: cycle threshold

	Initial variant(s)		Alpha variant		Delta variant		Omicron variant	
	Value	CI	Value	CI	Value	CI	Value	CI
Mean	28.6005	(27.4128, 29.7366)	27.5550	(26.8631, 28.1723)	25.3484	(24.1440, 26.4360)	27.9723	(27.2051, 28.6550)
Median	28.7700	(28.0900, 30.7000)	27.1000	(26.0800, 28.1100)	25.1800	(23.8000, 26.4800)	28.3550	(26.6528, 29.6502)
Skewness	-0.3298	(-0.5826, -0.0741)	0.0182	(-0.1801, 0.1442)	0.0812	(-0.1558, 0.3427)	-0.0404	(-0.2225, 0.1212)
Kurtosis	-0.5794	(-0.9539, -0.0888)	-0.8419	(-1.0267, -0.5896)	-0.8956	(-1.1593, -0.5571)	-1.1835	(-1.3132, -1.0312)

Figure 3E shows normalized values of the mean, median, skewness, and kurtosis for comparison purposes, presented through a radar chart. Results suggest that the highest viral loads, as inferred from the distribution of Ct values, are associated with the Delta variant period, showing a statistically significant difference from the others (p=0.0005), confirmed by the Kruskal-Wallis analysis and Dunn's post-hoc analysis. The remaining variants show no statistically significant distribution differences. 

In addition, the skewness and kurtosis of patients' Ct values were calculated across the four periods of study. The Delta variant exhibited the highest skewness (γ_1,delta_=0.08), followed by the Alpha variant (γ_1,alpha_=0.02), with the Omicron and initial variants showing negative skewness (γ_1,omicron_=-0.04 and γ_1,initial_=-0.33, respectively) (Figure 3C). Thus, the mean, median, and skewness assessments rank the four pandemic periods in the same order, regarding viral loads, confirming that mean, median, and skewness are each critical to interpreting and evaluating pandemic progression. Consequently, lower mean and median values correspond to greater skewness values, indicating an inverse relationship between them. Specifically, lower mean and median and higher skewness of Ct values correlate with greater disease severity and pandemic progression, as detailed in the following sections. Conversely, the kurtosis evaluation did not yield comparable conclusions. 

In Figure 3D, the relationship between the median and skewness of Ct values is illustrated, with the reproduction rate and positivity rate values represented through a color code for each month. Each oval corresponds to a month (from mid-2020 to mid-2022) and is positioned on the graph according to the Ct values it represents. The median axis has been inverted to enhance the visualization of the correlation. Spearman's correlation coefficient revealed a very strong correlation between mean and median Ct values (rho=0.98; p<0.001) and a moderate positive correlation between mean Ct values and the positivity rate (rho=0.48; p=0.02). The relationship between median and skewness was found to be negative, with a Spearman coefficient of rho of -0.48 (p=0.02), while no statistically significant correlation was found between the median and the reproduction rate.

Ct and pandemic evolution

**Figure 4:**
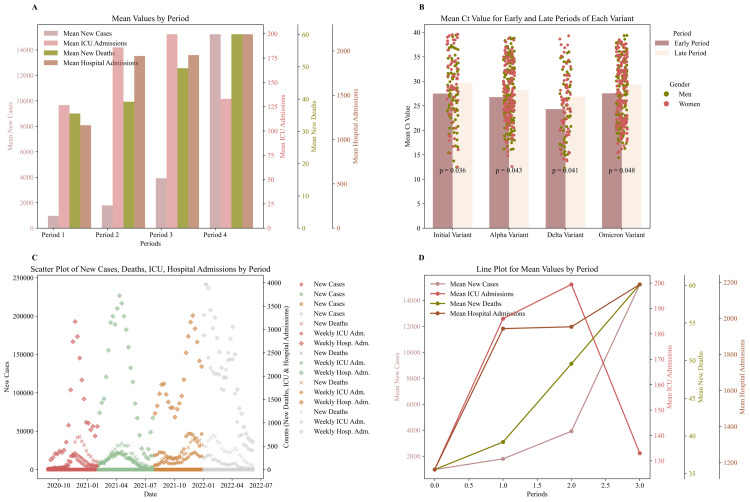
COVID-19 indicator trends and Ct values across pandemic periods (A) The plot presents the means for new cases (left axis), ICU admissions, new deaths, and hospital admissions across four defined COVID-19 pandemic periods, with separate axes for each metric to facilitate comparison of health indicator fluctuations. (B) The bar charts depict the mean Ct values of the rising and declining phases (early and late periods) of the pandemic per wave/variant. The dot plots show the gender information, along with p-values from the Ct value comparison. (C) The scatter plots show the new cases, new deaths, weekly ICU admissions, and weekly hospital admissions for each period (initial, Alpha, Delta, Omicron). Each period is presented in a different color. (D) The line plot illustrates the fluctuations in the mean values of COVID-19 indicators across the four periods. Ct: cycle threshold; ICU: intensive care unit

**Figure 5:**
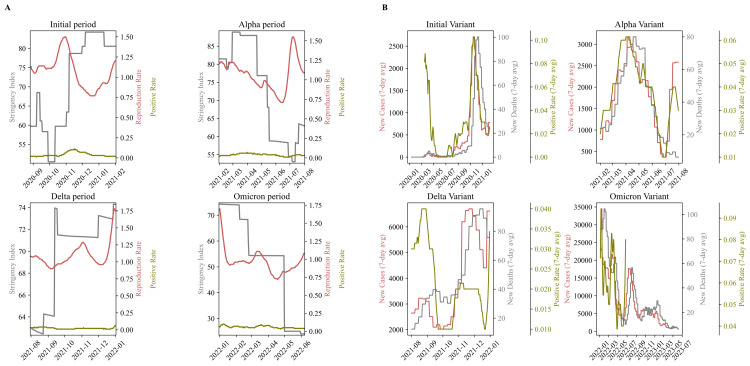
Epidemiological metrics across pandemic periods (A) The subplots consist of four individual plots, each representing a different period of the pandemic. In each plot, the left axis shows the stringency index, while the right axis displays the positivity rate and reproduction rate. (B) The positivity rate, new cases, and new deaths (seven-day average) are shown for the four study periods, highlighting peak values as well as growth and decline phases. New cases are displayed on the left axis, while the right double axis represents new deaths and the positivity rate.

The evolution of the Ct values, which is inversely related to viral load, appears to be directly correlated with the progression of the pandemic in the country. By dividing the study period into four main waves, we investigated whether increases in cases, deaths, and positivity rates aligned with higher viral load values and whether decreases in these metrics coincided with a decline in the pandemic wave. Each period was further divided into two sub-periods: one corresponding to an increase in cases and the other to a decrease. The transition point between these sub-periods was approximately determined by the peak in cases and deaths (Figure 4B-4C and Figure 5B).

As shown in Figure 4B, higher Ct values were observed during periods of declining cases, with statistical significance (p<0.05) across all four study periods. During periods of rising cases, higher viral loads (lower Ct values) were observed, while lower viral loads were noted during declining phases. This study revealed that significantly higher viral loads were associated with periods of rising cases, whereas lower viral loads were associated with periods of decline. These findings suggest that patient viral load may provide insights into the progression of pandemic waves.

Additionally, when analyzing all waves of the pandemic, we observed that during the dominance of the Omicron variant, the ​​lowest reproduction rate was recorded (mean R=0.96<1) along with simultaneously low viral load values (Table 2 and Figure 2C). During this period, restrictive measures were relaxed (mean strictness index=53.52). Despite the relaxed measures, the maximum positivity rate was recorded, implying increased cases, hospitalizations, and deaths. Given that this period does not align precisely with a specific calendar range, these results may reflect the high transmissibility of this variant. The high transmissibility of the variant is underscored by the fact that the reproduction rate was below 1, which would typically correspond to a decrease in cases. In contrast, during the Omicron period, the highest positivity rates were observed, followed by the initial and Alpha variants, with the Delta variant showing the lowest rates (Figure 5A-5B and Table 2).

**Table 2:** Indicators and metrics of the pandemic evolution ICU: intensive care unit

	Initial period		Alpha		Delta		Omicron	
	Mean	Range	Mean	Range	Mean	Range	Mean	Range
Stringency index	71.30	50.46-84.26	72.92	53.01-88.89	69.53	62.45-74.31	53.52	24.07-74.28
Positivity rate	0.04	0.01-0.11	0.04	0.01-0.07	0.02	0.01-0.06	0.06	0.03-0.1
Reproduction rate	1.07	0.77-1.5	1.07	0.71-1.55	1.09	0.9-1.78	0.96	0.72-1.7
Weekly hospital admissions	1173	208-3167	1943	266-3723	1953	1123-3299	2188	596-3964
Weekly ICU hospital admissions	127	18-354	186	13-389	199	96-367	133	19-312

Ct and disease severity 

Since lower Ct values imply a higher viral load, we investigated whether higher viral loads are associated with more severe disease. These lower Ct values, linked to higher viral loads, may correlate with the increased severity observed during the Delta variant period. During the Delta variant period, which showed the highest viral loads, there was also a significant increase in the number of hospitalizations relative to the positivity rate. 

According to the SEIR model, developed and fitted with Greek data, as presented in the publication by Liossi et al. [17], the proportion of infected individuals requiring hospitalization was 3.8% during the Delta variant period, decreasing to 2.18% during the period when the Omicron variant was predominant in the country. Additionally, data from [18] showed that the number of intubated patients per day was 438 during the Delta wave, falling to 395 during the Omicron wave. 

The mean weekly number of hospitalizations in ICUs, during the Omicron period, was significantly lower compared to the period when the Alpha and Delta variants prevailed in the country. Specifically, weekly hospitalizations were reduced by 28% and 33% during the Omicron period compared to Alpha and Delta (Figure 4C-4D and Table 2). This potentially highlights the milder disease caused by the Omicron variant, possibly due to population immunity or the intrinsic characteristics of the virus. Conversely, during the Delta period, the lowest positivity rate (PR_mean_=2%) and the highest mean weekly hospitalizations were observed (Table 2), which may underscore the greater disease severity associated with the Delta variant. 

To draw conclusions regarding disease severity during the four study sub-periods, hospitalization rates for patients in regular hospital beds or ICUs were calculated. Rates were calculated based on the positivity rate, rather than new cases, to yield a more accurate ratio during periods of fluctuating case reporting as shown in the following equations: $\text{Hospitalization rate} = \frac{\text{Hospitalizations}}{\text{Positive rate}} \times 100$ (Equation 1), $\text{ICU rate} = \frac{\text{ICU hospitalizations}}{\text{Positive rate}} \times 100$ (Equation 2), and $\text{Hospitalization index} = \sqrt{\text{Hospitalization rate} \times \text{ICU rate}}$ (Equation 3). Additionally, the geometric mean of hospitalization rates (hospitalization index) in regular beds and ICUs was calculated, as this measure is considered more reliable for representing overall trends in rate- or ratio-based variables.

**Figure 6:**
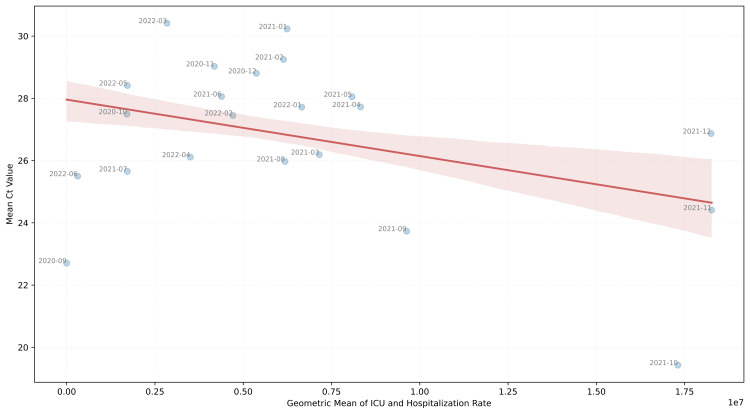
Relationship between ICU and hospitalization rates and mean Ct values The scatter plot depicts the relationship between the geometric mean of ICU and hospitalization rates and the mean Ct value per month. Each data point represents a monthly average, annotated with the respective month's date. The red line represents the regression line, indicating the linear relationship between the variables, and the shaded area shows the confidence interval around the regression line. Ct: cycle threshold; ICU: intensive care unit

The calculations were conducted both monthly and across the prevalence periods of each variant. During the period when the Delta variant prevailed, the hospitalization index was calculated as 1.4×10⁷, for the Alpha variant 5.99×10⁶, for the Omicron variant 3.92×10⁶, and for the initial variants 3.5×10⁶. Therefore, based on the statistics presented in Table 2, combined with the proposed hospitalization index metric, it was found that the Delta variant, followed by the Alpha, Omicron, and lastly initial variants, posed a progressively greater risk for severe disease. The negative relationship between the metrics suggests that as ICU admissions and hospitalizations increase, the mean Ct value decreases, indicating a general inverse trend between the two metrics (Figure 6).

These data may indicate a correlation between Ct values and disease severity. If viral load is indeed related to disease severity, it could serve as a biomarker for predicting patient progression. Combined with other patient parameters, machine learning and statistical models could be developed to predict the disease severity and assist in formulating strategies and implementing and adapting therapeutic protocols.

## Discussion

Understanding the molecular nature of the virus and the mechanisms affecting the interaction between SARS-CoV-2 and the target cells is critical for developing mathematical models to explain the differences between its variants [19]. Extensive genomic analyses have shown that the initial SARS-CoV-2 virus strain is a beta-CoV genus. Phylogenetic homology with BatCoVRaTG13 type has been detected [20,21]. The SARS-CoV-2 genome consists of 29,903 nucleotides. The RNA-dependent RNA polymerase (Rd-Rp) enzyme is responsible for intracellular virus replication in target epithelial cells. Morphologically, the spherical domain of the SARS-CoV-2 virion consists of the following four main proteins: spike surface glycoprotein (S), matrix or main protein (M), envelope protein (E), and nucleocapsid protein (NC). Additionally, 16 non-structural proteins (NSP1-NSP16) have been recognized. These proteins are responsible for encoding critical virus molecules, such as helicase and RNA-directed RNA polymerase, that trigger virus replication and translation in the ribosome machinery [22]. A small number of accessory proteins (ORF3a-ORF8) have also been identified, but their activity remains under investigation [23,24]. The S glycoprotein projections are the main characteristic of the virion's surface consisting of two subunits, the S1/S2, which create a crown-like formation. S1 is the main receptor-binding domain (RBD), whereas S2 is involved in the fusion mechanism between the cell membrane and virions. Furin, trypsin, cathepsin, and serine protease (transmembrane serine protease 2 (TMPRSS2)) molecules are involved in the virus cell entry process enhancing the intracellular infection signal [25-27]. The virus-cell interaction is mediated by the human angiotensin-converting enzyme 2 (hACE2), which is the prominent functional receptor for SARS-CoV-2, leading to successive cell membranous attachment and entry, activating the S1 and S2 subunit complex [20,28]. Regarding the clinical impact of the different variants (Alpha, Beta, Delta, Omicron, etc.) on patients, significant clinico-molecular analyses have revealed a strong relationship between specific genetic characteristics of the virus, viral load, and the transmission dynamics of COVID-19 [28,29].

Based on these molecular characteristics, the present study examines how viral load, as reflected by Ct values, varies across different SARS-CoV-2 variants and pandemic waves. In particular, it focuses on the viral load analysis of the specific Alpha, Delta, and Omicron SARS-CoV-2 variants in a broad pool of COVID-19 patients categorized in the pandemic waves. The data indicated that there is no significant correlation between viral load and demographic characteristics such as age and gender in COVID-19 patients. Analysis of Ct values across different COVID-19 variants revealed distinct statistical characteristics. The study found statistically significantly lower mean and median Ct values, along with increased skewness, during the Delta variant period compared to other variants, underscoring the importance of these measures for understanding pandemic dynamics. The findings suggest that higher viral loads correlate with rising case numbers, while lower viral loads align with declines in pandemic waves. In addition, lower Ct values, which indicate higher viral loads, may also be associated with greater disease severity. Notably, during the Delta variant period, hospitalizations increased markedly relative to the positivity rate. When calculating the geometric mean of hospitalizations in regular beds and ICUs, a negative relationship was observed between Ct values and the geometric mean of hospitalizations. This implies that higher hospitalization rates may correlate with higher viral load measurements, potentially indicating greater disease severity in hospitalized patients. The Omicron variant, despite showing lower reproduction rates and viral load, exhibited the highest positivity rates and hospitalizations, possibly due to its high transmissibility. These findings suggest that viral load may serve as a biomarker for predicting disease severity and the evolution of pandemic waves. 

In summary, Ct values may serve as a useful and easily accessible indicator for epidemiological surveillance. They may also inform public health decisions related to resource management and the implementation of control measures. Overall, the results highlight that Ct values offer valuable insights for epidemiological monitoring and intervention strategies.

Limitations

The study's conclusions are based solely on observations from data collected at NIMTS. Therefore, larger-scale studies, such as those at the national or international level, are necessary to validate the results. Readers should take into account that Ct value measurements present uncertainties, regarding, for instance, the collection technique, the specimen type, and the sampling time [10,30]. In this study, PCR tests were conducted using three different analyzers, so the measurement errors are not consistent among analyzers and could influence the results. Consequently, potential sources of bias include both selection and measurement bias. The analysis of Ct values should take into account potential variations caused by technical factors, such as the characteristics of the PCR analyzer, as well as by epidemiological conditions, such as the viral variant. In this study, a stratified analysis was applied for each viral variant. In addition, non-parametric methods were used to reduce the influence of potential distributional irregularities caused by measurement variability. These limitations are consistent with those reported in similar studies (e.g., [30]), which suggests that the use of complementary indicators (e.g., the ratio of viral to human RNA in the sample) could further support comparability between Ct measurements. Furthermore, in some analyses, the p-value was calculated to be close to the predefined significance level of 5%. If a stricter significance level, such as 1%, had been used, the results would not have been considered statistically significant. Consequently, validating these findings by increasing the sample size is crucial.

## Conclusions

The understanding of viral load behavior at the intra-host level, as well as the differences observed among variants or subvariants, can be used to draw conclusions regarding disease progression in patients and the evolution of the pandemic. Viral load, as measured by Ct values, may serve as a useful indicator of disease severity and transmission dynamics. The study revealed that the lowest Ct values were observed during periods of increased pandemic waves, whereas higher Ct values were recorded as the pandemic waves declined. Moreover, the wave with the lowest Ct values also corresponded to the highest number of hospitalizations and deaths. Overall, higher viral loads were associated with more severe disease and the growth of pandemic waves. In Greece, both trends were observed during the period when the Delta variant prevailed. Leveraging Ct values can improve predictions, simplify the modeling process, and contribute to the design of pandemic mitigation strategies. The evolution of Ct values could serve as a reliable indicator of pandemic trends and support decision-making regarding the implementation of interventions and the allocation of healthcare resources. Prospective, multicenter studies are needed to validate these findings, assess their generalizability, and address limitations related to measurement and selection bias. Incorporating clinical outcomes into future research will also be important for confirming these conclusions.
